# Characteristics of dental fear among Arabic-speaking children: a descriptive study

**DOI:** 10.1186/1472-6831-14-118

**Published:** 2014-09-22

**Authors:** Azza A El-Housseiny, Najlaa M Alamoudi, Najat M Farsi, Douaa A El Derwi

**Affiliations:** Paediatric Dentistry Department, Professor of Paediatric Dentistry, Faculty of Dentistry, King Abdulaziz University, Jeddah, Saudi Arabia; Paediatric Dentistry Department, Professor of Paediatric Dentistry, Faculty of Dentistry, Alexandria University, Alexandria, Egypt; Head of Paediatric Dentistry Department, Faculty of Dentistry, King Abdulaziz University, Jeddah, Saudi Arabia; Paediatric Dentistry Department, Professor of Public Health and Community Medicine, Faculty of Dentistry, King Abdulaziz University, Jeddah, Saudi Arabia; Public Health and Community Medicine Department, Faculty of Medicine, Cairo University, Cairo, Egypt

**Keywords:** Child dental fear, Anxiety, Arabic version, CFSS-DS, Factor analysis, Assessment

## Abstract

**Background:**

Dental fear has not only been linked to poor dental health in children but also persists across the lifespan, if unaddressed, and can continue to affect oral, systemic, and psychological health. The aim of this study was to assess the factor structure of the Arabic version of the Children’s Fear Survey Schedule-Dental Subscale (CFSS-DS), and to assess the difference in factor structure between boys and girls.

**Methods:**

Participants were 220 consecutive paediatric dental patients 6–12 years old seeking dental care at the Faculty of Dentistry, King Abdulaziz University, Saudi Arabia. Participants completed the 15-item Arabic version of the CFSS-DS questionnaire at the end of the visit. Internal consistency was assessed using Cronbach’s alpha. Factor analysis (principal components, varimax rotation) was employed to assess the factor structure of the scale.

**Results:**

The Cronbach’s alpha was 0.86. Four factors with eigenvalues above 1.00 were identified, which collectively explained 64.45% of the variance. These factors were as follows: Factor 1, ‘fear of usual dental procedures’ consisted of 8 items such as ‘drilling’ and ‘having to open the mouth’, Factor 2, ‘fear of health care personnel and injections’ consisted of three items, Factor 3, ‘fear of strangers’, consisted of 2 items. Factor 4, ‘fear of general medical aspects of treatment’, consisted of 2 items. Notably, four factors of dental fear were found in girls, while five were found in boys.

**Conclusions:**

Four factors of different strength pertaining to dental fear were identified in Arabic-speaking children, indicating a simple structure. Most items loaded high on the factor related to fear of usual dental procedures. The fear-provoking aspects of dental procedures differed in boys and girls. Use of the scale may enable dentists to determine the item/s of dental treatment that a given child finds most fear-provoking and guide the child’s behaviour accordingly.

**Electronic supplementary material:**

The online version of this article (doi:10.1186/1472-6831-14-118) contains supplementary material, which is available to authorized users.

## Background

Fearful children visit the dentist less regularly and have more dental caries compared to non-fearful children [1]. In addition, child dental fear and the tendency for parents to avoid bringing their fearful child to dental visits were among the risk indicators for caries in children [2]. Moreover, it has been reported that 61% of children with dental fear exhibit difficult-to-manage behaviour during dental appointments [3], and these children may require special behaviour guidance strategies such as treatment with nitrous oxide [4].

Fear in children can be measured by many methods. These include physiological measurements, observational methods, and psychometric assessments, such as the Children’s Fear Survey Schedule-Dental Subscale (CFSS-DS [5]).

The CFSS-DS was developed in 1982 to assess dental fear in children; it includes 15 items related to dental treatment such as ‘drilling’, ‘having to open the mouth’, and ‘injections’. Either the child’s parent or the child rates each item on a 5-point Likert scale that ranges from 1 (‘not afraid at all’) to 5 (‘very afraid’), yielding a total score that ranges from 15 to 75 [5]. Many reviews have shown that the CFSS-DS is the most commonly used psychometric method, which is approaching the gold standard, for measuring fear in paediatric dental research [6–9]. Out of all the scales used for children, it is the only one that has been translated into a number of different languages [8]. The CFSS-DS shows high internal consistency, high test re-test reliability, and adequate construct and criterion validity in English as well as in several other languages [5, 10–14].

While few studies have been conducted to assess the structure of the CFSS-DS, the existing data suggest that the CFSS-DS items fit a three-factor structure, and this structure most sensitively reflects child dental fear in some populations (e.g. Western cultures) [15, 16]. However, in other populations, such as Bosnian children and Chinese children in Canada, four factors were identified [14, 17].

Evidence regarding differences in dental fear between boys and girls has been inconsistent: some investigators report that girls are more fearful [11, 12], while others found no significant difference between boys and girls [13, 16]. It is unclear whether the CFSS-DS structure differs between boys and girls. Investigations in the Netherlands [16] and India [13] suggested gender differences in CFSS-DS structure, with four factors identified for girls and three factors identified for boys. It has been suggested by ten Berge et al. [16], that the gender differences found in their study population should be further explored.

It has been suggested that normative data regarding dental fear should be collected from children of different cultures, since the development of dental fear may be affected by cultural and social habits, as well as the dental care system of the country [7]. Arabic countries, in particular, have cultural and social habits that differ from those of other countries; such cultural characteristics include the differential treatment of sons and daughters in Arabic families. For example, parents encourage boys to be brave and behave like men, but are overprotective of girls. These rearing patterns may result in different CFSS-DS factor structure for boys versus girls. Since norms are not available for the Arabic version of the CFSS-DS, studies assessing dental fear in Arabic- speaking children are needed. Thus, it is necessary to investigate the characteristics of the CFSS-DS in Arabic-speaking children. Early recognition of dental fear in children is important to deliver effective treatment using the appropriate behaviour guidance strategies.

The aim of this study was to assess the factor structure of the Arabic version of the Children’s Fear Survey Schedule-Dental Subscale (CFSS-DS) and to assess the difference in factor structure between boys and girls.

## Methods

### Subjects

Over a period of 8 months (Sept 2011–April 2012), 220 consecutive healthy 6- to 12-year-old paediatric dental patients seeking treatment at the paediatric dental clinics in the Faculty of Dentistry, King Abdulaziz University, Saudi Arabia, participated in this study. The sample size was determined as the minimal number for reliable results for factor analysis should be more than 100 (in each gender) and 5 times the number of items [18]. To allow for incomplete questionnaires and other unforeseen problems the sample was increased to 110 participants for each gender. Inclusion criteria were as follows: (1) healthy (normal with no organic, physiologic, biochemical, psychiatric, mental, or communication disorders); (2) aged 6–12 years; (3) of any Arabic nationality; (4) the primary and native language of the child and parent was Arabic; (5) the child and/or his/her parent could read Arabic.

### Questionnaire and procedure

The Arabic version of the CFSS-DS was adapted from the English version [5]. The scale consists of 15 items related to various aspects of dental treatment, such as ‘the dentist drilling’, ‘injections’, and ‘having to open the mouth’ (Additional file 1).

The questionnaire was translated into formal Arabic language by a native speaker, and corrections in the Arabic translation were made according to the results of a pilot study. The clarity of item 12 (‘choking’) was improved through the addition of ‘entrance of something into the throat’. In item 15, the word ‘nurse’ was replaced by ‘dentist’ because dentists clean patients’ teeth in Arabic communities. The questionnaire was then translated back into English by another person and compared to the English version to confirm that they matched [19].

**Figure 1:**
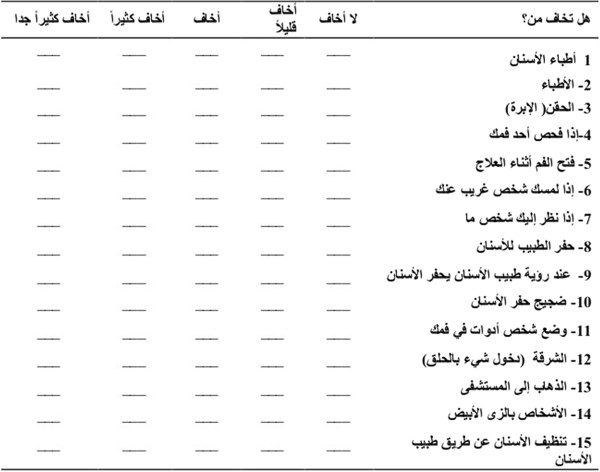
**The Arabic version of children’s fear survey schedule-dental subscale.**

Immediately following a participant’s dental visit, a trained receptionist presented him or her with the Arabic version of the 15-item questionnaire (Figure 1) to complete. Participation was voluntary. The objectives of the study as well as the questionnaire were explained to the parent, and written consent was obtained. Children were instructed to rate the degree to which they feared each item on the questionnaire by giving a score from 1 to 5. A higher score indicated greater fear. Parents of children who could not yet read were instructed to help the child with reading, but to refrain from influencing his/her responses. The receptionist observed questionnaire completion to ensure parents did not influence the children’s responses but only read it. Personal data were obtained from the parents of the children.

Ethical approval was obtained from the Research Ethics committee, Faculty of Dentistry, King Abdulaziz University. Informed consent was obtained from the parents and verbal approval from the children.

### Statistical methods

Data were analysed using SPSS version 18.0 (SPSS Inc., Chicago, IL, USA). Descriptive statistics and t-tests were used to compare age and total CFSS-DS score according to gender. Cronbach’s Alpha was used to test internal consistency. Significance level was set at P < 0.05.

To assess the reliability and validity of a psychometric scale, it is usually subjected to internal consistency and test-retest reliability, criterion validity, construct validity, and factor analysis [20]. Our previous study reported that the Arabic version of the CFSS-DS had high internal consistency (Cronbach’s alpha = 0.86), high test-retest reliability (intra-class correlation = 0.86, P < 0.001), and acceptable construct and criterion validity [i.e. significant correlations using Spearman’s rho were found between total fear score and both willingness to return to the dentist (r = 0.50, p < 0.001) and the Frankl behaviour rating scale (r = -0.54, p < 0.001)] [19].

### Principal component analysis

In this study, factor analysis (Principal Component, varimax rotation) was employed to assess the factor structure of the Arabic version of the CFSS-DS. Factor analysis uses the correlation matrix between items on a scale to determine whether a subset of items is related in such a way that suggests that they are measuring the general concept of interest [20] (e.g. dental fear). Principal Component Analysis extracts factors and retains the maximum amount of common variance possible in the first factor. Subsequent factors retain the maximum amount of the remaining common variance until all common variance is included [18]. Factors are always listed in descending order according to the amount of variation they explain [i.e. from the highest (first factor) to the lowest (last factor)]. An eigenvalue indicates the amount of variance explained by each factor. Eigenvalues above 1.00 are considered strong enough to be retained [18]. Within each factor, item loading was categorised as follows: >0.70 excellent, >0.63 very good, >0.55 good, >0.45 fair, and >0.32 poor [21]. For each item, the highest loading in a factor was taken into account.

The Kaiser-Meyer-Olkin measure (KMO) was used to determine sampling adequacy. A KMO value equal to 0.70 indicates that factor analysis can be performed.

## Results

Of the 220 children, one girl was excluded because of missing data (she returned the questionnaire without any answer). The mean age of the children was 8.97 ± 1.76 years. Boys comprised 50.22% of the sample and had a mean age of 8.99 ± 1.88 years; girls constituted 49.77% of participants and had a mean age of 8.96 ± 1.63 years. No significant difference was found in age between boys and girls (P = 0.89).

Total fear scores ranged from 15–57, with a mean value of 23.00 ± 7.75. The total mean CFSS-DS score was 23.50 ± 7.66 for boys and 23.51 ± 7.85 for girls, with no statistically significant difference observed between the two groups (P = 0.23). The most feared items, in descending order, were ‘injections’, ‘the dentist drilling’, ‘choking’, and ‘having a stranger touch you’. However, the ranking of these items differed between boys and girls (Table 1).

**Table 1:** **CFSS-DS mean item scores and standard deviation (SD) for all children, boys and girls**

Item	All (N = 219)		Boys		Girls	
	Mean	SD	Mean	SD	Mean	SD
1. Dentists	1.42	0.79	1.36	0.78	1.47	0.81
2. Doctors	1.34	0.69	1.34	0.76	1.35	0.61
3. Injections (shots)	2.33	1.33	2.18	1.13	2.49	1.32
4. Having somebody examine your mouth	1.22	0.56	1.20	0.49	1.24	0.62
5. Having to open your mouth	1.30	0.74	1.28	0.73	1.32	0.76
6. Having a stranger touch you	1.78	1.05	1.65	0.93	1.91	1.16
7. Having somebody look at you	1.62	0.98	1.49	0.83	1.75	1.10
8. The dentist drilling	1.90	1.15	1.87	1.15	1.93	1.16
9. The sight of the dentist drilling	1.65	1.05	1.65	1.07	1.65	1.02
10. The noise of the dentist drilling	1.60	0.97	1.54	0.94	1.66	1.01
11. Having somebody put instruments in your mouth	1.58	0.97	1.51	0.90	1.64	1.04
12. Choking	1.85	1.13	1.90	1.24	1.81	1.00
13. Having to go to the hospital	1.21	0.59	1.25	0.61	1.16	0.56
14. People in white uniforms	1.08	0.39	1.10	0.47	1.06	0.30
15. Having the dentist clean your teeth	1.20	0.62	1.21	0.65	1.19	0.59

CFSS-DS: The Children’s Fear Survey Schedule-Dental Subscale.

The nationality distribution of the children was as follows: 45.66% Saudi, 28.77% Yemeni, 10.50% Palestinian, 5.94% Egyptian, 4.66% Sudanese, 1.37% Jordanian, and 3.20% other Arabic nationality.

### Principal component analysis

Cronbach’s alpha was 0.86. The KMO value was 0.85. The factor structure after varimax rotation is shown in Table 2. There were four factors with eigenvalues above 1.00, which collectively accounted for 64.45% of the variance. These factors were as follows: Factor 1, ‘fear of usual dental procedures’ consisted of 8 items, such as ‘drilling’ and ‘having to open the mouth’; Factor 2, ‘fear of health care personnel and injections’, consisted of 3 items; Factor 3, ‘fear of strangers’ consisted of 2 items; and Factor 4, ‘fear of general medical aspects of treatment’, consisted of 2 items.

**Table 2:** **Rotated CFSS-DS factor matrix for all the children (n = 219)**

Item	Factor 1	Factor 2	Factor 3	Factor4
1. Dentists	0.302	0.810*	-0.102	0.053
2. Doctors	0.010	0.754*	0.214	0.090
3. Injections	0.420	0.460*	0.232	0.073
4. Having somebody examine your mouth	0.592*	0.164	0.127	0.280
5. Having to open your mouth	0.668*	0.262	0.046	-0.096
6. Having a stranger touch you	0.046	0.070	0.877*	0.016
7. Having somebody look at you	0.133	0.109	0.844*	0.077
8. The dentist drilling	0.738*	0.324	0.036	0.136
9. The sight of the dentist drilling	0.755*	0.233	-0.058	0.052
10. The noise of the dentist drilling	0.878*	0.053	0.017	0.043
11. Having somebody put instruments in your mouth	0.789*	0.063	0.131	0.199
12. Choking	0.690*	0.069	0.048	0.152
13. Having to go to the hospital	0.125	0.260	0.069	0.830*
14. People in white uniforms	0.218	-0.060	0.022	0.818*
15. Having the dentist clean your teeth	0.652*	-0.155	0.184	0.268
**Eigen value**	5.499	1.616	1.333	1.221
**% of explained variance**	30.374	12.092	11.154	10.834

*The highest loading for each item is presented in bold face.

Factor 1: Fear of usual dental procedures.

Factor 2: Fear of health care personnel and injections.

Factor 3: Fear of strangers.

Factor 4: Fear of general medical aspects of treatment.

The KMO value was 0.76 for girls and 0.77 for boys. Some differences existed between girls and boys (Tables 3 and 4). For girls, a four-factor structure was found. Factor 1 was ‘fear of usual dental procedures and injections’ , and it consisted of 9 items. Each of the other three factors consisted of two items (Factor 2: ‘fear of strangers’ , Factor 3: ‘fear of general medical aspects of treatment’ , and Factor 4: ‘fear of health care personnel’). However, for boys, five factors were identified: Factor 1, ‘fear of usual dental procedures’ included 6 items; Factor 2, ‘fear of general medical aspects of treatment’ included 2 items; Factor 3, ‘fear of less invasive dental procedures’ , included 2 items; Factor 4, ‘fear of health care personnel and injections’ , included 3 items; and Factor 5, ‘fear of strangers’ , included 2 items.

**Table 3:** **Rotated CFSS-DS factor matrix for girls (n = 109)**

Item	Factor 1	Factor 2	Factor 3	Factor4
1. Dentists	0.281	0.084	0.469	0.657*
2. Doctors	0.177	0.185	-0.113	0.758*
3. Injections	0.475*	0.383	0.377	0.147
4. Having somebody examine your mouth	0.605*	0.117	0.176	0.267
5. Having to open your mouth	0.763*	0.046	0.058	0.277
6. Having a stranger touch you	0.035	0.893*	-0.058	0.060
7. Having somebody look at you	0.081	0.869*	0.048	0.105
8. The dentist drilling	0.728*	0.104	0.283	0.144
9. The sight of the dentist drilling	0.823*	-0.091	0.056	0.299
10. The noise of the dentist drilling	0.868*	-0.026	-0.094	0.100
11. Having somebody put instruments in your mouth	0.773*	0.080	0.189	0.138
12. Choking	0.696*	0.104	0.225	-0.278
13. Having to go to the hospital	0.138	-0.020	0.771*	0.251
14. People in white uniforms	0.072	-0.008	0.786*	-0.230
15. Having the dentist clean your teeth	0.589*	0.193	-0.042	-0.327
**Eigen value**	5.49	1.72	1.49	1.28
**% of explained variance**	31.38	12.21	12.05	10.87

*The highest loading for each item is presented in bold face.

Factor 1: Fear of usual dental procedures and injections.

Factor 2: Fear of strangers.

Factor 3: Fear of general medical aspects of treatment.

Factor 4: Fear of health care personnel.

**Table 4:** **Rotated CFSS-DS factor matrix for boys (n = 110)**

Item	Factor 1	Factor 2	Factor 3	Factor 4	Factor 5
1. Dentists	0.441	-0.121	0.064	0.757*	-0.194
2. Doctors	-0.166	0.220	0.166	0.826*	0.242
3. Injections	0.441	0.060	-0.007	0.545*	0.064
4. Having somebody examine your mouth	0.462*	0.439	0.320	0.011	0.107
5. Having to open your mouth	0.144	-0.049	0.882*	0.188	0.010
6. Having a stranger touch you	0.002	0.154	0.045	0.015	0.846*
7. Having somebody look at you	0.196	0.075	0.056	0.080	0.809*
8. The dentist drilling	0.788*	0.111	0.185	0.316	0.064
9. The sight of the dentist drilling	0.828*	0.131	-0.030	0.066	0.090
10. The noise of the dentist drilling	0.796*	0.194	0.396	-0.005	0.037
11. Having somebody put instruments in your mouth	0.608*	0.309	0.514	-0.100	0.163
12. Choking	0.643*	0.114	0.302	0.214	0.070
13. Having to go to the hospital	0.025	0.892*	0.107	0.180	0.197
14. People in white uniforms	0.316	0.771*	0.024	-0.022	0.058
15. Having the dentist clean your teeth	0.388	0.396	0.684*	0.019	0.117
**Eigen value**	5.65	1.79	1.50	1.05	1.01
**% of explained variance**	24.43	13.42	12.96	11.92	10.55

*The highest loading for each item is presented in bold face.

Factor 1: Fear of usual dental procedures.

Factor 2: Fear of general medical aspects of treatment.

Factor 3: Fear of less invasive dental procedures.

Factor 4: Fear of health care personnel and injections.

Factor 5: Fear of strangers.

## Discussion

In the present study the mean CFSS-DS score for all the children was 23.0 which falls in the range of fear scores (22.1 – 33.25) observed by previous studies [10–13, 15, 16]. However other studies reported higher mean fear scores of 37.8 and 45.9 for fearful children [10, 22]. No significant difference was found between boys and girls in the total fear scores that is supported by previous studies [13, 16, 22], however other studies done on schoolchildren reported that girls have more fear scores than boys [11, 12].

The Arabic version of the CFSS-DS has a high internal consistency (0.86), which is in accordance with previously reported values, which ranged from 0.83 to 0.92 across several different languages [11–15]. This indicates a high correlation between answers to different items on the scale [20].

In this study, factor analysis was performed on the Arabic version of the CFSS-DS to determine the factor structure of the instrument and to determine the factor accounting for the majority of the variance in dental fear, as measured by the CFSS-DS. Four factors were extracted: Factor 1, ‘fear of usual dental procedures’; Factor 2, ‘fear of health care personnel and injections’; Factor 3, ‘fear of strangers’; and Factor 4, ‘fear of general medical aspects of treatment’. This finding is consistent with those obtained in populations in Bosnia [14] and the United States (data reported by Alesalo et al. [15]), as well as in a population of Dutch children with dental fear [22]. In Finland [15], the Netherlands [16], Japan [11], and India [13], three factors were identified, and the total variance ranged from 54% to 65%.

The factor constructs found in the present study are relatively consistent with previously reported constructs; for example, ‘fear of general, less invasive dental treatment’ (Factor 1), ‘fear of medical aspects’ (Factor 2), ‘fear of drilling’ (Factor 3), and ‘fear of strangers’ (Factor 4) were observed in Dutch children with dental fear [22]. In Bosnia and Herzegovina, ‘fear of the usual dental practice’ (Factor 1), ‘fear of doctors and white uniforms’ (Factor 2), ‘fear of extreme situations’, ‘injections, choking, and having to go to the hospital’ (Factor 3), and ‘fear of items related to strangers’ (Factor 4) were found [14]. However, slight differences were observed in Finnish children [15], where the identified factors were ‘fear of highly invasive dental procedures’ (Factor 1), ‘fear of potential victimization, strangers, choking, and hospital’ (Factor 2), and ‘fear of less invasive procedures’ (Factor 3). Almost the same three factor constructs were found in Japan, but the rank order between the second and third factors differed [11]. However, three factors with different constructs were identified in India [13]. Variations in factor components among distinct populations may reflect cultural, social, and demographic differences. Dental care systems in developing countries, such as India [13], Bosnia [14], and some Arabic countries, often vary from those of Western, developed countries. For example, oral health education programs are deficient in developing nations. In addition, parents usually take their children to the dentist for treatment of oral diseases, but rarely for prevention. Moreover, some people may only seek dental treatment when in pain. These variables may influence children’s experience and perception of fear.

Although factor sequence, number, and content differed among studies, it was observed in the present study as well as in other studies [14, 16, 22] that most items loaded relatively highly on the first factor, indicating one primary dimension: ‘fear of dental treatment in general’ (found in the present study, in Dutch children with dental fear [22], and in Bosnian children [14]) or ‘fear of highly invasive dental procedures’ (found in children in Japan, Finland, and the Netherlands [11, 15, 16]). As ten Berge et al. [16] concluded, the CFSS-DS essentially measures ‘a one-dimensional concept’ of dental fear.

Notably, although ‘injections’ received one of the highest fear rankings by children in this study as well in other studies [5, 11, 15, 22], ‘injections’ did not load heavily on Factor 1 and only moderately loaded (0.46) on Factor 2 (‘fear of health care personnel and injections’). This is consistent with findings of other studies where ‘injections’ loaded strongly (0.61) with Factor 2, ‘fear of medical aspects’, in Dutch children with dental fear [22], or had a good loading (0.55) with Factor 3, ‘fear of extreme situations’ (e.g., injections, choking, and having to go to the hospital), in Bosnia [14]. This may be explained by the fact that fear of injections is more closely associated with medical rather than dental treatment, since paediatric dentists do not allow the child to see the injection syringe. Moreover, paediatric dentists use distraction and substituted words, such as ‘sleepy juice’, when administering dental anaesthesia to children to keep them from realizing they are receiving an injection. In addition, some authors reported that general dentists may not regularly use local anaesthesia during restoration, or may only use hand instruments, when treating children [3, 23]. In addition, factor analysis of general fear scales suggests that dental fears are related more to lack of control than to \components of medical treatment, such as injection and injury [24].

The small difference in factor pattern between different studies, including the present study, seems to indicate that children differentiate between aspects of dental treatment. This is supported by the findings of ten Berge et al. [22], which indicated that separate concepts might exist within the general concept of dental fear. In the present study, part of the children’s fear may have been associated with a fear of doctors, dentists, and injections (Factor 2); this may be attributable to parenting in the Arabic communities, as some parents use the threat of injections, doctors, or dentists as a method of child discipline. ‘Fear of strangers’ (Factor 3) was identified as an important component of the children’s fear. This finding supports those of previous studies in which items related to strangers and being touched or looked at comprised one factor with [11, 15, 22] or without items related to choking [14, 17].

In the present study ‘fear of strangers’ was located in Factor 2 in girls while, it was located in boys in Factor 5. Similar results were found in the Netherlands, where items related to strangers comprised a separate factor for Dutch girls, but not boys [16]. This indicates that fear of strangers plays a greater role in the fear construct for girls. This gender difference reflects the differences between girls and boys in responding to fearful aspects of dental treatment. Thus, the components of dental visits preferentially aversive to each gender should be considered during dental treatment and discussed in dental education.

This study has several limitations. In previous studies the questionnaire is often completed before treatment. This is contrary to the CFSS-DS design as it is supposed to be filled after treatment to avoid the false results as a child may express anticipatory anxiety prior to treatment [25]. Order effects may have affected participant responding on the CFSS-DS. Administering the questionnaire after a dental procedure may have directed participants to report less dental fear, as significant reduction in dental fear after treatment was found by Klaassen et al. [26] (CFSS-DS score was 45.1 before treatment and dropped to 32.2 after treatment). Type of dental procedure performed may have an impact on participants response. However, dental treatment with or without local anesthesia was not related to the CFSS-DS scores [12, 19]. The sample was obtained from a clinical university context, which limits the generalizability of the present findings. Further studies on more representative samples of schoolchildren are necessary to understand dental fear in children who do not go to dentists and those of different socioeconomic levels. Additional studies are needed to evaluate fear in children with behaviour problems. In order to provide further evidence for the validity of the Arabic version of the scale, the scale should be compared with other self-report measures that assess dental fear in children. Future research is also needed on samples obtained from schools in different Arabic countries. In addition, future studies should include more subjects to be able to study factor structure according to different age groups.

In the Middle East, there are 22 Arabic countries containing more than 360 million people. In addition to immigration, Arabic peoples mingle with other countries worldwide as a result of scholarship, diplomatic positions, and other missions. These individuals usually relocate with their family and children to a foreign country and live there for several years. During this period, their children may require dental treatment; thus, it would be beneficial if dental practitioners worldwide were knowledgeable of the fear norms among Arabic-speaking children. However, there is a lack of Arab participation in research about dental fear in children. This study provides the Arabic version of the CFSS-DS, which has not been previously presented in the literature. Through use of this version in further research, fear norms in Arabic-speaking children can be identified, and Arabic participation in fear research can be enriched.

Although differences in cultures exist, the CFSS-DS appears to be able to measure different aspects of dental fear in Arabic-speaking children. Children can differentiate between different items pertaining to dental fear. By using the CFSS-DS, the dentist may recognize which item(s) of the scale reported by the child are related to a particular child’s fear. Accordingly, the dentist can approach the child and guide his/her behaviour during the dental visit. It is recommended that this scale be used as a basic evaluation tool in addition to other assessment tools, such as the caries risk assessment and dietary assessments, for paediatric patients.

## Conclusions

Four factors of different strength pertaining to dental fear were identified in Arabic-speaking children, indicating that the Arabic version of the CFSS-DS has a simple factor structure. Most items loaded high on the factor related to fear of usual dental procedures. The fear-provoking aspects of dental procedures differed in boys and girls. Use of the scale may enable dentists to determine the item/s of dental treatment that a given child finds most fear-provoking and guide the child’s behaviour accordingly.

## Electronic supplementary material

Additional file 1: **The children’s fear survey schedule-dental subscale.** (PDF 140 KB)

## Abbreviations

CFSS-DS: The children’s fear survey schedule-dental subscale
KMO: Kaiser-meyer-olkin test.
