# Supplementary material for: Characteristics of dental fear among Arabic-speaking children: a descriptive study
Source: BMC Oral Health. 2014 Sep 22;14:118. doi: 10.1186/1472-6831-14-118 (PMC4180733; doi:10.1186/1472-6831-14-118)
Supplement: Supplementary file 1 — Additional file 1: The children’s fear survey schedule-dental subscale. (PDF 140 KB) [file 12903_2014_448_MOESM1_ESM.pdf]

Children's Fear Survey Schedule-Dental Subscale					
	**Not afraid at all**	**A little afraid**	**A fair amount afraid**	**Pretty much afraid**	**Very afraid**
1. Dentists	____	____	____	____	____
2. Doctors	____	____	____	____	____
3. Injections	____	____	____	____	____
4. Having someone examine your mouth	____	____	____	____	____
5. Having to open your mouth	____	____	____	____	____
6. Having a stranger touch you	____	____	____	____	____
7. Having somebody look at you	____	____	____	____	____
8. The dentist drilling	____	____	____	____	____
9. The sight of the dentist drilling	____	____	____	____	____
10.The noise of the dentist drilling	____	____	____	____	____
11. Having somebody put instruments in your mouth	____	____	____	____	____
12. Choking	____	____	____	____	____
13. Having to go to the hospital	____	____	____	____	____
14. People in white uniforms	____	____	____	____	____
15. Having the nurse clean your teeth	____	____	____	____	____
